# Associations between household environmental factors and immature mosquito abundance in Quetzaltenango, Guatemala

**DOI:** 10.1186/s12889-019-8102-5

**Published:** 2019-12-23

**Authors:** Zachary J. Madewell, Silvia Sosa, Kimberly C. Brouwer, José Guillermo Juárez, Carolina Romero, Audrey Lenhart, Celia Cordón-Rosales

**Affiliations:** 1 Centro de Estudios en Salud, Universidad del Valle de Guatemala, Guatemala City, Guatemala; 2 Program in Public Health (Epidemiology), University of California, San Diego/San Diego State University, San Diego, CA USA; 3 Department of Family Medicine & Public Health, Division of Global Health, University of California, San Diego, CA USA; 4 Department of Entomology, Texas A&M University, College Station, TX USA; 5 Centers for Disease Control and Prevention (CDC), Atlanta, GA USA

**Keywords:** Immature mosquito abundance, Mosquito larvae, Mosquito pupae, Arbovirus vectors, Household environment, Proximity to houses, Proximity to roads, Quetzaltenango, Guatemala, Central America

## Abstract

**Background:**

*Aedes aegypti*-borne diseases are becoming major public health problems in tropical and sub-tropical regions. While socioeconomic status has been associated with larval mosquito abundance, the drivers or possible factors mediating this association, such as environmental factors, are yet to be identified. We examined possible associations between proximity to houses and roads and immature mosquito abundance, and assessed whether these factors and mosquito prevention measures mediated any association between household environmental factors and immature mosquito abundance.

**Methods:**

We conducted two cross-sectional household container surveys in February–March and November–December, 2017, in urban and rural areas of Quetzaltenango, Guatemala. We used principal components analysis to identify factors from 12 variables to represent the household environment. One factor which included number of rooms in house, electricity, running water, garbage service, cable, television, telephone, latrine, well, and sewer system, was termed “environmental capital.” Environmental capital scores ranged from 0 to 5.5. Risk factors analyzed included environmental capital, and distance from nearest house/structure, paved road, and highway. We used Poisson regression to determine associations between distance to nearest house/structure, roads, and highways, and measures of immature mosquito abundance (total larvae, total pupae, and positive containers). Using cubic spline generalized additive models, we assessed non-linear associations between environmental capital and immature mosquito abundance. We then examined whether fumigation, cleaning containers, and distance from the nearest house, road, and highway mediated the relationship between environmental capital and larvae and pupae abundance.

**Results:**

We completed 508 household surveys in February–March, and we revisited 469 households in November–December. Proximity to paved roads and other houses/structures was positively associated with larvae and pupae abundance and mediated the associations between environmental capital and total numbers of larvae/pupae (*p* ≤ 0.01). Distance to highways was not associated with larval/pupal abundance (*p* ≥ 0.48). Households with the lowest and highest environmental capital had fewer larvae/pupae than households in the middle range (*p* < 0.01).

**Conclusions:**

We found evidence that proximity to other houses and paved roads was associated with greater abundance of larvae and pupae. Understanding risk factors such as these can allow for improved targeting of surveillance and vector control measures in areas considered at higher risk for arbovirus transmission.

## Background

Approximately 6.01 billion people currently live in areas suitable for *Aedes aegypti* disease transmission [1]. *Ae. aegypti*-borne diseases, such as dengue (DENV), chikungunya (CHIKV), and Zika (ZIKV) viruses, are found in tropical and subtropical zones with an abundance of these species, including Central America [2–4]. Other than for yellow fever vaccine [5], no broadly licensed commercial vaccines are available for the principal *Ae. aegypti*-borne arboviruses, so vector control remains the primary strategy to limit their spread [6]. Climate change, urbanization, migration, human behavior, and ecosystem modification are among the myriad factors influencing the geographic spread of *Ae. aegypti* and their associated viruses [1, 7, 8].

*Ae. aegypti* are highly productive in urban environments and have a strong preference for human blood [9]. *Ae. aegypti* spend the majority of their lives in the houses where they emerged, flying an average of 40–80 m during the course of their lifetimes [10]. Oviposition sites are selected based on their physical, chemical, and biological characteristics, such as container type, depth, water quality, and sun exposure [11, 12]. Ideal larval habitats for *Ae. aegypti* are dark-colored containers filled with stagnant water and organic material in shaded areas around houses [11, 13, 14]. Productive container types include flower pots, tires, vases, buckets, cans, rain gutters, fountains, bottles, and birdbaths [11, 13, 14]. Greater human population densities provide more feeding opportunities for *Ae. aegypti* [15]*.*

Studies of socioeconomic status (SES) impacts on *Ae. aegypti* abundance mostly report greater *Ae. aegypti* population densities in low SES areas [16–22]. Most studies have only considered income, occupation, and education as the SES factors. Few studies have evaluated associations between household environmental measures as attributes of SES and mosquito abundance. The household environmental factors that can influence mosquito infestation are quite heterogeneous. These include piles of garbage [21], open wells [23, 24], storm sewers [25], and septic tanks [26]. Less information is available on spatial risk factors, but proximity to vacant lots [27, 28], vegetation or green spaces [29], other houses/structures [30], and roads [31, 32], have been shown to be predictive of mosquito abundance. Household infrastructure may also influence the mosquito microenvironment [33–35]. For example, the *premise condition index* has been shown to be an effective tool at classifying houses according to risk of having mosquito breeding sites [33–35]. This index can be used to prioritize neighborhoods for vector control interventions.

For this study, we evaluated whether proximity to other houses/structures and roads, and household environmental factors were associated with immature mosquito abundance. A secondary objective was to determine how mosquito abatement interventions, including fumigation and cleaning possible larval habitat containers, influence immature mosquito abundance. It is particularly important to examine these relationships in Central America, which has been host to large outbreaks of arbovirus infection and where vector control resources are limited [36].

## Methods

### Study site

**Fig. 1:**
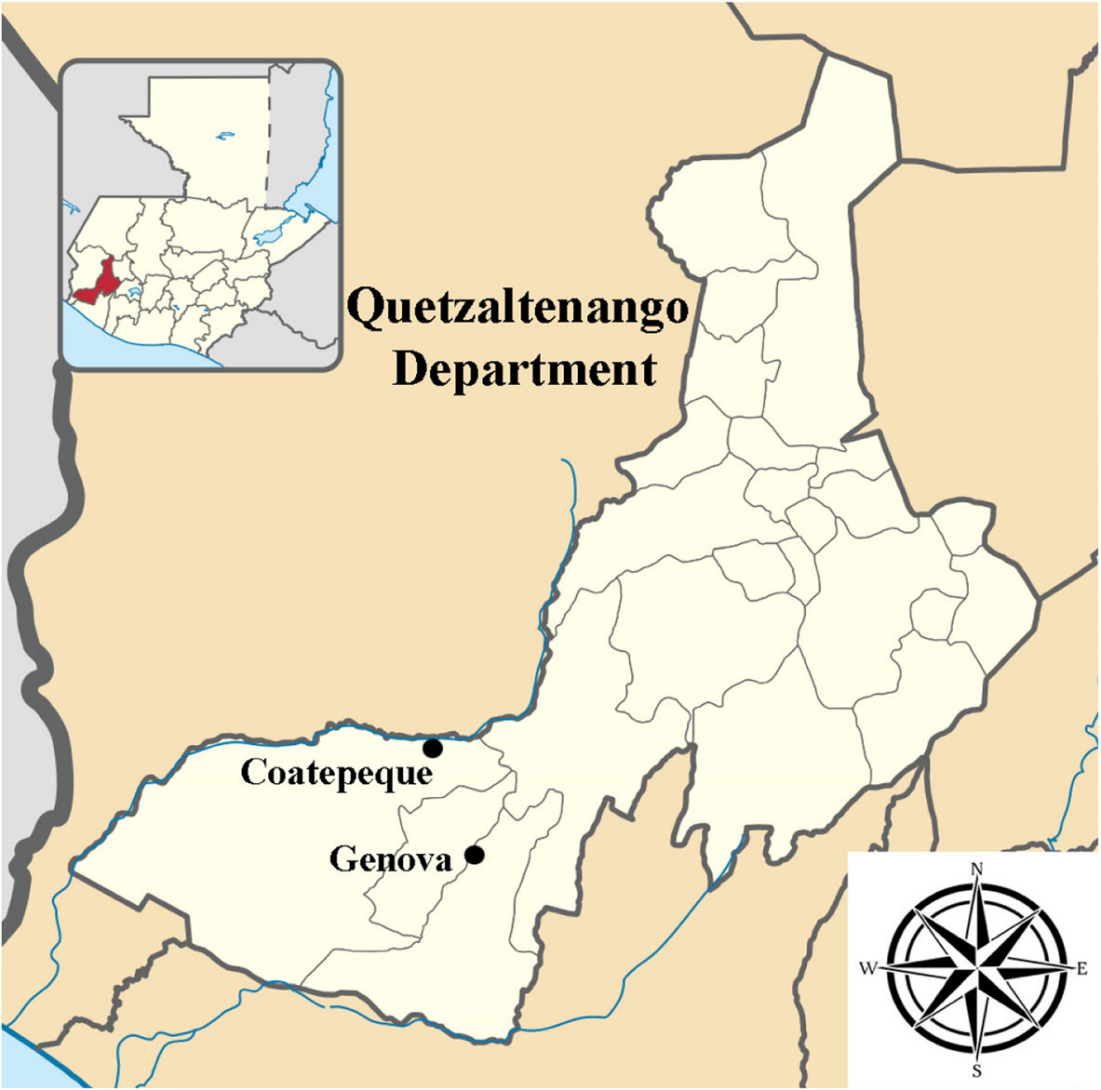
Coatepeque and Génova, Quetzaltenango Department, Guatemala. Source: Quetzaltenango department location map; by user Edouno; licensed under CC BY 3.0 via Wikimedia Commons, https://commons.wikimedia.org/wiki/File:Quetzaltenango_department_location_map.svg

We selected two municipalities in the Guatemalan department of Quetzaltenango, Coatepeque and Génova (Fig. 1), as study sites based on their high risk for arboviral disease transmission and high mosquito pupal index (> 25% of houses with pupal infestations) [37]. Coatepeque (14°42′00″N 91°52′00″O) and Génova (14°37′00″N 91°50′00″O) are located in the south-western region of the Republic of Guatemala and have a tropical climate. The mean annual temperatures for Coatepeque and Génova are 25.7 °C and 26.2 °C, respectively, the mean annual precipitations are 308 mm and 285 mm, and the mean elevations are 498 m and 350 m [38]. This study included two communities in Coatepeque (La Unión, El Jardín) and six communities in Génova (30 de Junio, Robles, Nueva Italia, Génova, San Jose, Guadalupe) (Additional file 1: Figure S1). The communities in Coatepeque were selected based on the presence of *Vigilancia Integrada Comunitaria* (Integrated Community Surveillance), a prospective public health syndromic surveillance system for diarrheal, respiratory, and febrile illnesses of the Centro de Estudios en Salud/Universidad del Valle de Guatemala in collaboration with the Guatemalan Ministry of Health and the United States Centers for Disease Control and Prevention (CDC). In Génova, all of the communities reporting a high pupal index were included, with the exception of one community that posed a security risk for field personnel. Six sites in Génova were selected to achieve comparable population size to the two sites in Coatepeque. We remotely identified each probable house structure within each community using Google satellite imagery for 2016 in QGIS 2.2 (QGIS Development Team, 2019). The Ministerio de Salud Pública y Asistencia Social (MSPAS) provided detailed maps of each community in order to demonstrate community boundaries. All probable houses were identified and verified on-site to confirm classification of structures [39]. Houses were then randomly selected in each village using a two-stage sampling procedure based on a geographic 100 × 100 m grid. We first randomly selected grids, enumerated households, and then used a random number generator to select one house within each grid. In both Coatepeque and Génova, selected houses accounted for 10% of the total community population (*n* = 250 and *n* = 258, respectively). If no one was at home during recruitment, if householders chose not to participate, or if the selected structure was not a house, we selected the nearest house to the right of the front door as a replacement.

### Container inspection and questionnaire

After obtaining informed consent from homeowners, we conducted cross-sectional surveys for container-inhabiting mosquitoes in February–March, 2017 (the local dry season) and November–December, 2017 (the local rainy season) in both Génova and Coatepeque. We conducted two surveys to capture immature mosquito abundance in Guatemala’s two seasons. All containers ≥3 L inside and outside the houses were inspected for any genera of mosquito larvae and pupae, and total numbers of mosquito larvae and pupae from all containers in each house and the containers with any mosquito larvae or pupae were recorded. Larvae and pupae were analyzed separately, as pupal counts are considered more representative of local adult mosquito populations [40, 41]. We did not identify larval and pupal genus or species. We interviewed the heads of household or another adult residing in the house, and responses were transcribed onto Excel spreadsheets. Questions covered mosquito control measures, waste disposal, and socioeconomic indicators.

### Variables

We assessed household environment factors and distance from nearest house/structure, paved road, and main transportation corridor running through the city/village as risk factors for vector concentrations. We assumed the main transportation corridor was the nearest highway or the only paved road in villages that did not have highway access.

We used principal components factor analysis to identify factors based on 12 variables from the first household survey to represent household attributes of SES. These included: number of rooms in the house (1–4, > 5), electricity (yes, no), running water (yes, no), a television (yes, no), a landline telephone (yes, no), a latrine (yes, no), cable television service (yes, no), a mobile phone (yes, no), trash disposal service (yes, no), a water well (yes, no), sewer system (yes, no), and a rainwater collection system (yes, no). The resultant compound factor, which we termed “environmental capital,” included all of the variables except a mobile phone and rainwater collection system (Additional file 1: Table S1). Variables highly correlated with the factor were weighted against their eigenvector. This factor reflects some of the attributes of the Encuesta Nacional de Salud Materno Infantil (National Survey of Maternal and Child Health), which focuses on the health of children and adults in Guatemala [42]. This household factor from the first survey explained 32% of the variability in the data and was used to represent environmental capital in the second survey as well. Higher environmental capital scores indicated higher SES and ranged from 0 to 5.5.

The measures of immature mosquito abundance were the total number larvae (continuous), total number of pupae (continuous), and positive containers (continuous). Categorical covariates included survey period (February–March vs. November–December), residence (urban vs. rural), self-reported cleaned (scrubbed, treated, or emptied standing water) containers (barrels, pots, tires, etc.) at least once in the last 6 months (yes, no), and self-reported homeowner or vector control authority fumigation inside/outside house at least once in the last 6 months (yes, no). Continuous covariates included the number of people in a household and the total number of containers ≥3 L with water at the time of the visit per household (e.g., buckets, barrels, flower pots, etc.). ‘Urban’ residences were those in El Jardín, Coatepeque, whereas ‘rural’ residences were all other communities, as defined by the census [43].

### Spatial analysis

Coordinates of each house were entered into geographical information system software (ArcGIS Pro 2.2.4 software; ESRI, Redlands, CA) and overlaid on basemaps and satellite images from December 8, 2018, of Coatepeque and Génova [44]. These maps were used to locate and visualize households and roads. We collected ground truth data through site visits during both survey periods. The distance between a house and its closest neighboring house or other structure (e.g., store, church) or road was ascertained by measuring the Euclidean distance between points taken from the front door of the house to the closest edge of lines representing roads [45–47]. Within the sub-set of sampled houses in each community, we also attempted to detect spatial clusters of houses with larval infestations.

### Statistical analysis

Medians and interquartile ranges were reported for continuous variables (total number of larvae; total number of pupae; number of positive containers; number of containers ≥3 l; number of people in household; distance to nearest paved road, highway, and house/structure; environmental capital). Frequency distributions were reported for categorical variables (cleaned containers, fumigation, urban/rural residence).

We used Poisson regression, which is used to model count data, to analyze unadjusted (Model 1) and adjusted (Model 2) associations between hypothesized risk factors (distance to nearest house/structure, paved road, highway), and immature mosquito abundance (number of larvae, pupae, and positive containers), with household as a repeated measure (two time points). We used generalized estimating equations to estimate the population-averaged effect and used compound symmetry as the covariance structure to account for correlations resulting from two measurements (February–March, November–December) of immature mosquito abundance on the same houses within each site. In Model 2, we used directed acyclic graphs [48, 49] to select each covariate for model inclusion based on a priori importance and evidence from the scientific literature of being potential confounders of associations between our exposures of interest and mosquito larvae and pupae abundance (Additional file 1: Figure S2). The adjusted models included environmental capital (categorized by tertiles) [50, 51], survey period [52], urban/rural residence [53], the number of people per household [54], cleaned containers [55], fumigated inside/outside house [56], and the total number of containers ≥3 l per household [50, 57]. Tolerance values were used to assess potential collinearity between all independent variables [58]. Due to the potential over-dispersion of larvae and pupae abundance, negative binomial regression models were fitted to evaluate the same associations as a sensitivity analysis [59].

We then used cubic spline generalized additive models to explore potential non-linear relationships between environmental capital and immature mosquito abundance (number of larvae, pupae, and positive containers) separately for both survey periods.

Finally, we assessed whether factors including fumigation, cleaned containers, and distance to nearest paved road, highway, and household/structure mediated the relationship between environmental capital and the total number of larvae, pupae, and positive containers. This analysis followed causal mediation analysis methods as previously described by VanderWeele [60]. The mediation models were Poisson models to estimate the association between environmental capital and the distance to the nearest house/structure, paved road, and highway, and binomial models to estimate the association between environmental capital and cleaned containers and fumigation history, which are dichotomous variables. The outcome models were Poisson models that estimated the association between environmental capital and immature mosquito indicators (number of larvae, pupae, and positive containers), adjusting for the mediators. All hypothesized mediators were included in outcome models. The “mediation” package in R 3.5.2 statistical software (R Development Core Team, Vienna, Austria) was used for multilevel causal mediation analyses [61]. We ran one thousand Monte Carlo simulations in this analysis for variance estimation. Estimates, standard errors, and the proportion mediated were reported. All analyses, other than mediation, were calculated using SAS V.9.4 (SAS Institute, Inc., Cary, North Carolina).

## Results

### Household characteristics

In February–March, 508 household inspections were completed. In November–December, 469 of those households (92.3%) were revisited for a second survey (some houses were not revisited because the homeowner was unavailable). An additional 18 households that were eligible but unavailable during the first survey were included in the second survey. Of all houses, 72.7% were in rural areas (Table 1). There was a median of five people per household. The median distances to the nearest house/structure, paved road, and highway were 3.1 m, 13.9 m and 244.1 m for rural residences and 1 m, 4.9 m, and 144.3 m for urban residences, respectively. The median numbers of larvae, pupae, and positive containers were 8, 1, and 1 in rural residences and 20, 2, and 1 in urban residences, respectively.

**Table 1:** Household characteristics and immature mosquito numbers, Coatepeque and Génova, Guatemala, 2017

Continuous variables	Median (IQR)
Number of people living in household	5 (4–6)
Total number of containers ≥3 L per household	4 (3–5)
Number of positive containers ≥3 L per household	1 (0–2)
Number of larvae in all containers ≥3 L per household	8 (0–50)
Number of pupae in all containers ≥3 L per household	1 (0–6)
Distance to nearest paved road (m)	9.5 (3.3–28.1)
Distance to nearest highway (m)	211.3 (57.4–404.3)
Distance to nearest building (m)	2 (1.0–5.3)
Environmental capital^a^	3.1 (1.8–4.1)
Categorical variables	% (SE)
Cleaned containers around house in previous 6 months	53.8 (1.6)
Fumigated inside or outside house in previous 6 months	30.1 (1.5)
Rural residence	72.7 (1.4)

In February–March, 508 surveys were completed. In November–December, 469 of those households were revisited for a second survey (92.3%). At that time, an additional 18 households were surveyed

*IQR* interquartile range, *SE* standard error

^a^Environmental capital was derived from principal components factor analysis and included: number of rooms in the household; presence of electricity, running water, a television, a landline telephone, cable, trash disposal, and sewer system; and absence of a water well and pit latrine. Score range: 0–5.5

### Geographical distances

Distance to the nearest paved road was inversely associated with the total number of larvae, pupae, and positive containers per house in Models 1 and 2 (*p* ≤ 0.01) (Table 2). For every 10-m increase in distance from the nearest paved road, the total number of larvae and positive containers decreased by a factor of 0.96 and the number of pupae decreased by a factor of 0.93, adjusting for environmental capital, urban/rural residence, the number of people per household, cleaned containers, fumigation history, and the total number of containers. Tolerance values were above 0.50, so there was no evidence of collinearity among any of the independent variables.

**Table 2:** Associations between geographical distances to roads/structures and immature mosquito abundance, Poisson regression, Coatepeque and Génova, Guatemala, 2017

Variable	Total number of larvae per household			Total number of pupae per household			Number of positive containers per household		
	β	SE	*P*-value	β	SE	*P*-value	β	SE	*P*-value
Distance from nearest paved road (10-m increase)									
Unadjusted	−0.04	0.01	< 0.01	− 0.06	0.03	< 0.01	− 0.05	0.01	< 0.01
Adjusted^a^	−0.04	0.01	< 0.01	−0.07	0.03	< 0.01	−0.04	0.01	< 0.01
Distance from nearest highway (100-m increase)									
Unadjusted	0.01	0.01	0.50	0.01	0.01	0.28	0.01	0.01	0.52
Adjusted^a^	0.01	0.01	0.55	0.01	0.01	0.35	0.01	0.01	0.47
Distance from nearest structure (1-m increase)									
Unadjusted	−0.03	0.01	< 0.01	−0.04	0.02	< 0.01	−0.04	0.01	< 0.01
Adjusted^a^	−0.03	0.01	< 0.01	−0.05	0.02	0.02	−0.03	0.01	< 0.01

^a^Adjusted for environmental capital, survey period, urban/rural residence, the number of people in a household, cleaned containers, fumigated inside or outside the house, and the total number of containers. Environmental capital was derived from principal components factor analysis and included: number of rooms in the household; presence of electricity, running water, a television, a landline telephone, cable, trash disposal, and sewer system; and absence of a water well and pit latrine

Distance to the nearest highway was not associated with the number of larvae, pupae, or positive containers per household in Models 1 and 2 (*p* ≥ 0.28) (Table 2).

Distance from the nearest household/structure was inversely associated with the total number of larvae and pupae and number of positive containers per house in Models 1 and 2 (*p* < 0.01) (Table 2). For every 1-m increase in distance from the nearest house/structure, the total number of larvae and positive containers decreased by a factor of 0.97 and the number of pupae decreased by a factor of 0.95, adjusting for relevant covariates. Full model outputs are reported in Additional file 1: Tables S2–S4, but these estimates should be interpreted with caution, because the relationships between the covariates and outcomes are not adjusted for confounders [49]. Results from negative binomial models were similar for distance to the nearest paved road, highway, and house/structure (Additional file 1: Table S5).

We did not verify measurements obtained using ArcGIS between houses and roads on the ground, but the ground resolution of the ArcGIS world imagery for our study sites is 0.46 m and objects in the map are within 5 m of their true location [62].

### Spatial clusters of larvae and pupae

High/Low clustering (Getis-Ord General G) analyses did not reveal spatially dependent clusters for immature mosquito abundance indicators (number of larvae, pupae, and positive containers) for either time point (*p* ≥ 0.40).

### Environmental capital

**Fig. 2:**
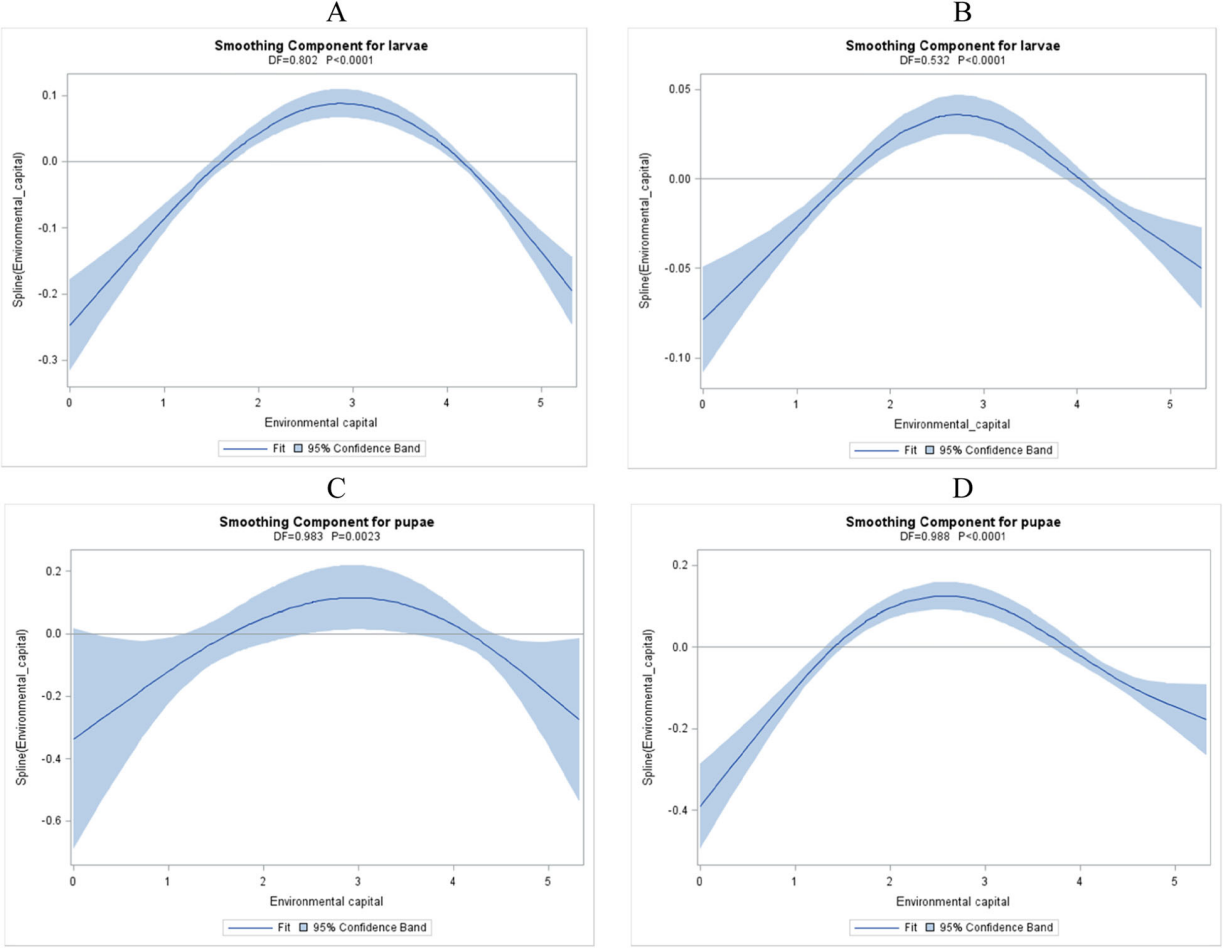
Cubic splines of associations between environmental capital and total number of larvae and pupae per household, Coatepeque and Génova, Guatemala, 2017. Panels A and B show results for larvae, whereas panels C and D show results for pupae. Panels A and C show results from the first survey in February–March, 2017, whereas Panels B and D show results from the second survey in November–December, 2017. The bands represent 95% confidence intervals

Cubic splines demonstrated significant non-linear relationships between environmental capital and the number of larvae and pupae per house that were similar for both survey periods (*p* < 0.01) (Fig. 2). For both surveys, households with the lowest and highest environmental capital had significantly fewer larvae and pupae compared to those in the middle (*p* < 0.01). Results for the number of positive containers were similar (Additional file 1: Fig. S3).

Distance to the nearest paved road and house/structure were significant mediators of the relationship between environmental capital and the number of larvae and pupae (*p* < 0.01) (Table 3). A one-unit increase in environmental capital was associated with a significant decrease in distance from the nearest paved road or house/structure, which in turn was associated with more larvae and pupae when environmental capital was held constant (*p* < 0.01). Fumigated houses, cleaned containers, and distance to the nearest highway were not significant mediators of the association between environmental capital and the number of larvae and pupae. Results for the number of positive containers were similar (Additional file 1: Table S6).

**Table 3:** Mediation of distances to roads/structures and mosquito prevention measures on the association between environmental capital and immature mosquito abundance, Coatepeque and Génova, Guatemala, 2017

Characteristic	Controlled direct effect		Natural indirect effect		Total effect		Proportion mediated
	Estimate	95% CI	Estimate	95% CI	Estimate	95% CI	
Total number of larvae							
Fumigated house	1.30*	1.12, 1.48	−0.14	−0.37, 0.10	1.16*	0.88, 1.45	−0.12
Cleaned containers	1.24*	1.06, 1.43	−0.12	−0.53, 0.28	1.12*	0.65, 1.56	−0.10
Distance to paved road (m)	−0.26*	−0.46, − 0.08	5.51*	5.15, 5.88	5.25*	4.95, 5.52	1.05
Distance to highway (m)	1.16*	0.98, 1.34	−0.03	− 0.05, 0.02	1.13*	0.94, 1.35	−0.03
Distance to nearest structure (m)	−0.01*	−0.23, − 0.19	3.22*	2.80, 3.65	3.21*	2.83, 3.59	1.00
Total number of pupae							
Fumigated house	0.01	−0.08, 0.10	−0.05	− 0.13, 0.03	−0.03	− 0.16, 0.09	0.73
Cleaned containers	−0.03	−0.13, 0.06	− 0.02	−0.08, 0.04	− 0.04	−0.17, 0.06	0.33
Distance to paved road (m)	−0.31*	−0.39, − 0.23	1.53*	1.39, 1.66	1.22*	1.11, 1.31	1.25
Distance to highway (m)	−0.03	−0.13, 0.06	− 0.01	−0.03, 0.02	− 0.04	−0.15, 0.08	0.18
Distance to nearest structure (m)	−0.31*	−0.42, − 0.21	0.85*	0.71, 1.01	0.55*	0.41, 0.68	1.56

*p-value< 0.05

## Discussion

This study identified environmental factors and SES attributes that were associated with mosquito larvae and pupae abundance. Distance to the nearest paved road and house/structure were inversely associated with larvae and pupae abundance and were significant mediators of the relationship between environmental capital and the number of larvae and pupae per house. Cubic splines revealed that households of middle environmental capital had significantly more larvae and pupae than those with the lowest and highest environmental capital.

Our finding that households closer to paved roads had more larvae and pupae is consistent with previous studies from Kansas and Bermuda, which found greater numbers of adult mosquitoes and eggs closer to roads [31, 32]. Proximity to paved roads may indicate greater population density, which would include more containers and greater availability of blood meals. The association remained significant after adjusting for the total number of containers ≥3 L per household, which may suggest a greater presence of smaller containers like cups, cans, and bottles, in areas closer to roads [31]. These containers are also conceivably productive larval habitats. This association was further supported by mediation analyses, which showed that distance to the nearest paved road was a significant mediator of the relationship between environmental capital and number of larvae and pupae. As environmental capital increased, distance to the nearest paved road decreased. Households closer to paved roads had significantly more larvae and pupae, holding environmental capital constant. It is conceivable that households with greater environmental capital, which are closer to roads, are more likely to own barrels and other large water storage containers, which may support larger mosquito populations if they are not properly managed. More mosquitoes in areas closer to paved roads may also increase the risk of the spread of arboviral infections, which was reported in a CHIKV study in Pakistan [63].

Distance to the nearest highway was not a significant predictor of larvae and pupae abundance. One study in Taiwan reported that the number of dengue fever cases corresponded inversely with distance from highways, further indicating that *Ae. aegypti* abundance may be associated with population density [64]. Proximity to highways in our study was not necessarily suggestive of greater human population density, which may have greater influence on mosquito abundance [65, 66]. These results may suggest that the immediate household environment contributes more to larvae and pupae abundance than more distant neighborhood factors [67–69]. This is particularly important for *Ae. aegypti*, as immatures tend to be highly aggregated in space and time, rarely dispersing beyond 30–40 m of the household where they developed as larvae [67, 69].

Distance to the nearest house/structure was inversely associated with larvae and pupae abundance. Furthermore, mediation analyses revealed that households with higher environmental capital were closer to other houses/structures and had significantly more larvae and pupae. We are unaware of other studies assessing distance to the nearest structure as a mediator between SES and mosquito abundance. Previous studies of associations between distance to the nearest building and mosquito abundance are inconsistent. Some report greater *Anopheles* and *Aedes* abundance in houses/structures closer together [30, 70, 71], whereas others do not [31, 72]. Urbanization and greater human population density lead to a greater number of artificial containers, which creates an abundance of potential habitats for mosquitoes, including tires, flowerpots, and cans [15]. Urban environments may also be more favorable for *Ae. aegypti* due to the absence of natural vegetation, competition, and predation [12, 15, 73, 74]. These results reinforce the premise that mosquito control requires community-wide efforts, as individual houses with disproportionately high numbers of mosquitoes may pose risks to their closest neighbors, and indeed the entire community [68].

Recent history of fumigation inside/outside of the house and containers that had been cleaned but could still serve as immature habitats for mosquitoes were not significant mediators between environmental capital and the number of larvae and pupae. Fumigating and cleaning containers with standing water are established mosquito control measures [55, 56, 75]. Fumigation is only provided by MSPAS in Guatemala. It could be that our measure of environmental capital was not predictive of these preventive measures in these communities or that fumigation may not have been effective in these areas. Alternatively, our cross-sectional survey that asked whether participants performed these prevention measures in the last 6 months may have been insufficient to assess the efficacy of these interventions, which require repeated application. Fumigation frequency and insecticide resistance should be also considered.

Households of middle environmental capital had significantly more larvae and pupae than households with the lowest and highest environmental capital for both surveys. In this study, environmental capital included access to running water, improved sanitation, a sewer system, and trash disposal service, which are typically associated with reduced mosquito populations [23, 24, 52, 76–78]. Greater environmental capital may also indicate higher values of other SES indicators, including income, occupation, and education, which are associated with greater mosquito prevention measures, such as removing containers with standing water [17–19, 21]. Conversely, low environmental capital was associated with greater distance to the nearest paved road, which was associated with fewer mosquitoes. It is conceivable that these distances exceeded the typical flight range for mosquitoes [79]. Moreover, houses with low environmental capital in this study had fewer barrels and other large water storage containers that were most productive for mosquitoes.

Our study did not characterize larval genus or species, but multiple species of *Aedes*, *Anopheles*, and *Culex* mosquitoes have been reported in Quetzaltenango department, where our study was conducted [80–83]. Specific species in Quetzaltenango include *Ae. aegypti* and *Ae. albopictus* [80, 81], which preferentially lay eggs in household containers [84]; *An. hectoris, An. parapunctipennis,* and *An. xelajuensis,* which prefer marshes, trees, swamps, fields, streams, and rivers [85]; and *Cx. corniger, Cx. peus,* and *Cx. quinquefasciatus*, whose breeding sites include storm sewers, cesspits, and polluted water [26, 86]. Given our container surveys occurred exclusively in households, we suspect that the majority of the immatures that we collected were either *Ae. aegypti* or *Ae. albopictus*.

Our study had several limitations. First, we sampled communities based on high entomological indices and are thus these are not representative of all communities in Guatemala. However, the households are representative of the local communities. Second, cross-sectional surveys of mosquitoes are time sensitive [41] and our two surveys points were insufficient to fully capture the temporal variability of mosquito larvae and pupae, despite including both dry and rainy seasons. Third, our survey assessments of whether participants fumigated inside/outside the house or cleaned their containers in the last 6 months were likely inadequate to assess the efficacy of these prevention strategies. Fourth, we did not include containers < 3 L on household premises such as discarded cups and cans, which could also serve as immature mosquito habitats.

## Conclusions

The global human population is expected to peak around 9.6 billion by 2050, favoring the spread of vector-borne diseases [87, 88]. With climate change, increasing temperatures, and more frequent flooding, the geographic range of *Ae. aegypti* and *Ae. albopictus* is increasing [1, 89]. The findings reported here provide evidence that proximity to other houses/structures and paved roads was associated with more mosquito larvae and pupae in containers around households. Furthermore, households with higher environmental capital were closer to other houses/structures and paved roads, and had significantly greater larvae and pupae abundance. Finally, households with middle environmental capital had significantly more larvae and pupae than the lowest and highest tiers. In resource-limited vector control programs, findings such as these can be used to focus efforts on areas of greater population density closer to roads. The findings also highlight the importance of programs that take into account neighborhood-level risks and mitigation strategies when promoting the prevention of vector-borne diseases.

## Supplementary information

**Additional file 1 Table S1.** Principal components factor analysis of household environment variables, Coatepeque and Génova, Guatemala, 2017 (*n* = 508). **Table S2**. Full model output of adjusted associations between geographical distances to paved roads and immature mosquito abundance, Poisson regression, Coatepeque and Génova, Guatemala, 2017. **Table S3**. Full model output of adjusted associations between geographical distances to highways and immature mosquito abundance, Poisson regression, Coatepeque and Génova, Guatemala, 2017. **Table S4**. Full model output of adjusted associations between geographical distances to houses or structures and immature mosquito abundance, Poisson regression, Coatepeque and Génova, Guatemala, 2017. **Table S5.** Associations between geographical distances to roads/structures and immature mosquito abundance, negative binomial regression, Coatepeque and Génova, Guatemala, 2017. **Table S6.** Mediation of distances to roads/structures and mosquito prevention measures on the association between environmental capital and the number of containers with any mosquito larvae or pupae per household, Coatepeque and Génova, Guatemala, 2017. **Figure S1.** Aerial view of communities in Coatepeque and Génova, Guatemala, 2017. **Figure S2**. Directed acyclic graphs of associations between geographical distances to roads and houses/structures and immature mosquito abundance. **Figure S3.** Cubic splines of associations between environmental capital and the number of containers with any mosquito larvae or pupae per household, Coatepeque and Génova, Guatemala, 2017.

## Data Availability

The surveys and data that support the findings of this study are not in English, but are available from the Centro de Estudios en Salud, Universidad del Valle de Guatemala, with permissions from the Centers for Disease Control and Prevention.

## Abbreviations

*Ae.*: *Aedes*
*An.*: *Anopheles*
CDC: Centers for Disease Control and Prevention
CHIKV: chikungunya virus
*Cx.*: *Culex*
DENV: dengue virus
IQR: interquartile range
MSPAS: Ministerio de Salud Pública y Asistencia Social
SE: standard error
SES: socioeconomic status
ZIKV: Zika virus

## References

[1] Ryan SJ, Carlson CJ, Mordecai EA, Johnson LR (2019). Global expansion and redistribution of Aedes-borne virus transmission risk with climate change. PLoS Negl Trop Dis.

[2] Pan American Health Organization (2018). Reported cases of dengue fever in the Americas: PLISA: health information platform for the Americas.

[3] Pan American Health Organization (2018). Zika suspected and confirmed cases reported by countries and territories in the Americas: cumulative cases, 2015–2018.

[4] Pan American Health Organization (2018). Chikungunya: data, maps and statistics.

[5] World Health Organization (2018). Yellow fever: key facts.

[6] Ritchie SA, van den Hurk AF, Smout MJ, Staunton KM, Hoffmann AA (2018). Mission accomplished? We need a guide to the ‘Post Release’World of Wolbachia for Aedes-borne disease control. Trends Parasitol.

[7] Kilpatrick AM, Randolph SE (2012). Drivers, dynamics, and control of emerging vector-borne zoonotic diseases. Lancet.

[8] Paixão ES, Teixeira MG, Rodrigues LC (2018). Zika, chikungunya and dengue: the causes and threats of new and re-emerging arboviral diseases. BMJ Glob Health.

[9] World Health Organization (2011). Comprehensive guideline for prevention and control of dengue and dengue haemorrhagic fever.

[10] Teurlai M, Menkes CE, Cavarero V, Degallier N, Descloux E, Grangeon J-P (2015). Socio-economic and climate factors associated with dengue fever spatial heterogeneity: a worked example in New Caledonia. PLoS Negl Trop Dis.

[11] Getachew D, Tekie H, Gebre-Michael T, Balkew M, Mesfin A (2015). Breeding sites of Aedes aegypti: potential dengue vectors in Dire Dawa, East Ethiopia. Interdiscip Perspect Infect Dis.

[12] Zahouli JB, Koudou BG, Müller P, Malone D, Tano Y, Utzinger J (2017). Urbanization is a main driver for the larval ecology of Aedes mosquitoes in arbovirus-endemic settings in South-Eastern Côte d'Ivoire. PLoS Negl Trop Dis.

[13] Centers for Disease Control and Prevention (2012). Dengue and the *Aedes aegypti* mosquito.

[14] Ferdousi F, Yoshimatsu S, Ma E, Sohel N, Wagatsuma Y (2015). Identification of essential containers for Aedes larval breeding to control dengue in Dhaka, Bangladesh. Trop Med Health.

[15] Li Y, Kamara F, Zhou G, Puthiyakunnon S, Li C, Liu Y (2014). Urbanization increases Aedes albopictus larval habitats and accelerates mosquito development and survivorship. PLoS Negl Trop Dis.

[16] Chambers D, Young L, Hill JH (1986). Backyard mosquito larval habitat availability and use as influenced by census tract determined resident income levels. J Am Mosq Control Assoc.

[17] Quintero J, Carrasquilla G, Suárez R, González C, Olano VA (2009). An ecosystemic approach to evaluating ecological, socioeconomic and group dynamics affecting the prevalence of Aedes aegypti in two Colombian towns. Cad Saude Publica.

[18] Unlu I, Farajollahi A, Healy SP, Crepeau T, Bartlett-Healy K, Williges E (2011). Area-wide management of Aedes albopictus: choice of study sites based on geospatial characteristics, socioeconomic factors and mosquito populations. Pest Manag Sci.

[19] Spiegel JM, Bonet M, Ibarra AM, Pagliccia N, Ouellette V, Yassi A (2007). Social and environmental determinants of Aedes aegypti infestation in Central Havana: results of a case–control study nested in an integrated dengue surveillance programme in Cuba. Tropical Med Int Health.

[20] Dhar-Chowdhury P, Haque CE, Lindsay R, Hossain S (2016). Socioeconomic and ecological factors influencing Aedes aegypti prevalence, abundance, and distribution in Dhaka, Bangladesh. Am J Trop Med Hyg.

[21] Syed M, Saleem T, Syeda U-R, Habib M, Zahid R, Bashir A (2010). Knowledge, attitudes and practices regarding dengue fever among adults of high and low socioeconomic groups. J Pak Med Assoc.

[22] Caprara A (2009). Lima JWdO, Marinho ACP, Calvasina PG, Landim LP, Sommerfeld J. irregular water supply, household usage and dengue: a bio-social study in the Brazilian northeast. Cad Saude Publica.

[23] Yamamoto S, Louis V, Sié A, Sauerborn R (2010). Household risk factors for clinical malaria in a semi-urban area of Burkina Faso: a case–control study. Trans R Soc Trop Med Hyg.

[24] Pålsson K, Jaenson TG, Dias F, Laugen AT, Björkman A (2004). Endophilic Anopheles mosquitoes in Guinea Bissau, West Africa, in relation to human housing conditions. J Med Entomol.

[25] Manrique-Saide P, Uc V, Prado C, Carmona C, Vadillo J, Chan R (2012). Storm sewers as larval habitats for Aedes aegypti and Culex spp. in a neighborhood of Merida, Mexico. J Am Mosq Control Assoc.

[26] Burke R, Barrera R, Lewis M, Kluchinsky T, Claborn D (2010). Septic tanks as larval habitats for the mosquitoes Aedes aegypti and Culex quinquefasciatus in playa-Playita, Puerto Rico. Med Vet Entomol.

[27] Baak-Baak CM, Arana-Guardia R, Cigarroa-Toledo N, Loroño-Pino MA, Reyes-Solis G, Machain-Williams C (2014). Vacant lots: productive sites for Aedes (Stegomyia) aegypti (Diptera: Culicidae) in Merida City, Mexico. J Med Entomol.

[28] Costa F, Fattore G, Abril M (2012). Diversity of containers and buildings infested with Aedes aegypti in Puerto Iguazú, Argentina. Cad Saude Publica.

[29] Estallo E, Sangermano F, Grech M, Ludueña-Almeida F, Frías-Cespedes M, Ainete M (2018). Modelling the distribution of the vector *Aedes aegypti* in a central Argentine city. Med Vet Entomol.

[30] Getis A, Morrison AC, Gray K, Scott TW (2003). Characteristics of the spatial pattern of the dengue vector, Aedes aegypti, in Iquitos, Peru. Am J Trop Med Hyg.

[31] Kaplan L, Kendell D, Robertson D, Livdahl T, Khatchikian C (2010). Aedes aegypti and Aedes albopictus in Bermuda: extinction, invasion, invasion and extinction. Biol Invasions.

[32] Ganser C, Wisely SM (2013). Patterns of spatio-temporal distribution, abundance, and diversity in a mosquito community from the eastern Smoky Hills of Kansas. J Vector Ecol.

[33] Andrighetti MTM, Galvani KC, da Graça Macoris ML (2009). Evaluation of premise condition index in the context of *Aedes aegypti* control in Marília, São Paulo, Brazil. Dengue Bull.

[34] Peres RC, Rego R, Maciel-de-Freitas R (2013). The use of the premise condition index (PCI) to provide guidelines for Aedes aegypti surveys. J Vector Ecol.

[35] Tun-Lin W, Kay B, Barnes A (1995). The premise condition index: a tool for streamlining surveys of Aedes aegypti. Am J Trop Med Hyg.

[36] Benelli G, Mehlhorn H (2016). Declining malaria, rising of dengue and Zika virus: insights for mosquito vector control. Parasitol Res.

[37] Ministerio de Salud Pública y Asistencia Social (2019). Sistema de Información Gerencial de Salud.

[38] Anacafe (2018). Estaciones meteorológicas - clima.

[39] Paquet C, Daniel M, Kestens Y, Léger K, Gauvin L (2008). Field validation of listings of food stores and commercial physical activity establishments from secondary data. Int J Behav Nutr Phys Act.

[40] Focks DA, Chadee DD (1997). Pupal survey: an epidemiologically significant surveillance method for Aedes aegypti: an example using data from Trinidad. Am J Trop Med Hyg.

[41] Morrison AC, Gray K, Getis A, Astete H, Sihuincha M, Focks D (2004). Temporal and geographic patterns of Aedes aegypti (Diptera: Culicidae) production in Iquitos, Peru. J Med Entomol.

[42] Ministerio de Salud Pública y Asistencia Social INdE, ICF International. Encuesta Nacional de Salud Materno Infantil 2014–2015: informe Final [Internet]. 2017.

[43] Instituto Nacional de Estadística - INE/Guatemala (2002). Catálogo de departamentos, municipios y lugares poblados de Guatemala (Censo 2002).

[44] ESRI (2018). World topographic map.

[45] Sander HA, Ghosh D, van Riper D, Manson SM (2010). How do you measure distance in spatial models? An example using open-space valuation. Environ Plann B: Plann Des.

[46] Mahabir R, Severson D, Chadee D (2012). Impact of road networks on the distribution of dengue fever cases in Trinidad, West Indies. Acta Trop.

[47] ESRI (Earth Science Resource Institute) (2019). How average nearest neighbor works.

[48] Weng H-Y, Hsueh Y-H, Messam LLM, Hertz-Picciotto I (2009). Methods of covariate selection: directed acyclic graphs and the change-in-estimate procedure. Am J Epidemiol.

[49] Westreich D, Greenland S (2013). The table 2 fallacy: presenting and interpreting confounder and modifier coefficients. Am J Epidemiol.

[50] Velasco-Salas ZI, Sierra GM, Guzmán DM, Zambrano J, Vivas D, Comach G (2014). Dengue seroprevalence and risk factors for past and recent viral transmission in Venezuela: a comprehensive community-based study. Am J Trop Med Hyg.

[51] Sissoko D, Moendandze A, Malvy D, Giry C, Ezzedine K, Solet JL (2008). Seroprevalence and risk factors of chikungunya virus infection in Mayotte, Indian Ocean, 2005-2006: a population-based survey. PLoS One.

[52] Fuller TL, Calvet G, Estevam CG, Angelo JR, Abiodun GJ, Halai U-A (2017). Behavioral, climatic, and environmental risk factors for Zika and Chikungunya virus infections in Rio de Janeiro, Brazil, 2015-16. PLoS One.

[53] Ali A, ur Rehman H, Nisar M, Rafique S, Ali S, Hussain A (2013). Seroepidemiology of dengue fever in Khyber Pakhtunkhawa, Pakistan. Int J Infect Dis.

[54] Vincenti-Gonzalez MF, Grillet M-E, Velasco-Salas ZI, Lizarazo EF, Amarista MA, Sierra GM (2017). Spatial analysis of dengue seroprevalence and modeling of transmission risk factors in a dengue hyperendemic city of Venezuela. PLoS Negl Trop Dis.

[55] Phuanukoonnon S, Mueller I, Bryan JH (2005). Effectiveness of dengue control practices in household water containers in Northeast Thailand. Tropical Med Int Health.

[56] Kenneson A, Beltrán-Ayala E, Borbor-Cordova MJ, Polhemus ME, Ryan SJ, Endy TP (2017). Social-ecological factors and preventive actions decrease the risk of dengue infection at the household-level: results from a prospective dengue surveillance study in Machala, Ecuador. PLoS Negl Trop Dis.

[57] Brunkard JM, López JLR, Ramirez J, Cifuefntes E, Rothenberg SJ, Hunsperger EA (2007). Dengue fever seroprevalence and risk factors, Texas–Mexico border, 2004. Emerg Infect Dis.

[58] Midi H, Sarkar SK, Rana S (2010). Collinearity diagnostics of binary logistic regression model. J Interdiscip Math.

[59] Lawless JF (1987). Negative binomial and mixed poisson regression. Can J Stat.

[60] VanderWeele T (2015). Explanation in causal inference: methods for mediation and interaction.

[61] Tingley D, Yamamoto T, Hirose K, Keele L, Imai K. Mediation: R package for causal mediation analysis. 2014.

[62] ESRI. ArcGIS Online Standard Service: World Imagery Collection, Map Server. Maps throughout this book were created using ArcGIS® software by ESRI. ArcGIS® and ArcMapTM Are the Intellectual Property of ESRI and Are Used Herein under License, Copyright ©ESRI. 2018. 2018 [cited 2019 April 28]. Available from: https://www.arcgis.com/index.html.

[63] Hira FS, Asad A, Farrah Z, Basit RS, Mehreen F, Muhammad K (2018). Patterns of occurrence of dengue and chikungunya, and spatial distribution of mosquito vector Aedes albopictus in Swabi district, Pakistan. Tropical Med Int Health.

[64] Hsueh Y-H, Lee J, Beltz L (2012). Spatio-temporal patterns of dengue fever cases in Kaoshiung City, Taiwan, 2003–2008. Appl Geogr.

[65] Obenauer JF, Joyner TA, Harris JB (2017). The importance of human population characteristics in modeling Aedes aegypti distributions and assessing risk of mosquito-borne infectious diseases. Trop Med Health.

[66] Padmanabha H, Durham D, Correa F, Diuk-Wasser M, Galvani A (2012). The interactive roles of Aedes aegypti super-production and human density in dengue transmission. PLoS Negl Trop Dis.

[67] LaCon G, Morrison AC, Astete H, Stoddard ST, Paz-Soldan VA, Elder JP (2014). Shifting patterns of Aedes aegypti fine scale spatial clustering in Iquitos, Peru. PLoS Negl Trop Dis.

[68] Yoon I-K, Getis A, Aldstadt J, Rothman AL, Tannitisupawong D, Koenraadt CJ (2012). Fine scale spatiotemporal clustering of dengue virus transmission in children and Aedes aegypti in rural Thai villages. PLoS Negl Trop Dis.

[69] Schafrick NH, Milbrath MO, Berrocal VJ, Wilson ML, Eisenberg JN (2013). Spatial clustering of Aedes aegypti related to breeding container characteristics in coastal Ecuador: implications for dengue control. Am J Trop Med Hyg.

[70] Minakawa N, Mutero CM, Githure JI, Beier JC, Yan G (1999). Spatial distribution and habitat characterization of anopheline mosquito larvae in western Kenya. Am J Trop Med Hyg.

[71] Mwangangi JM, Shililu J, Muturi EJ, Muriu S, Jacob B, Kabiru EW (2010). Anopheles larval abundance and diversity in three rice agro-village complexes Mwea irrigation scheme, Central Kenya. Malar J.

[72] Minakawa N, Sonye G, Yan G (2005). Relationships between occurrence of Anopheles gambiae sl (Diptera: Culicidae) and size and stability of larval habitats. J Med Entomol.

[73] Matysiak A, Roess A (2017). Interrelationship between climatic, ecologic, social, and cultural determinants affecting dengue emergence and transmission in Puerto Rico and their implications for Zika response. J Trop Med.

[74] Beaty BJ, Black W, Eisen L, Flores AE, García-Rejón JE, Loroño-Pino M (2016). The intensifying storm: domestication of *Aedes aegypti*, urbanization of arboviruses, and emerging insecticide resistance.

[75] Centers for Disease Control & Prevention (2018). Controlling mosquitoes at home.

[76] Nakkhara P, Chongsuvivatwong V, Thammapalo S (2013). Risk factors for symptomatic and asymptomatic chikungunya infection. Trans R Soc Trop Med Hyg.

[77] Aguiar BS, Lorenz C, Virginio F, Suesdek L, Chiaravalloti-Neto F (2018). Potential risks of Zika and chikungunya outbreaks in Brazil: a modeling study. Int J Infect Dis.

[78] Thai KT, Binh TQ, Giao PT, Phuong HL, Hung LQ, Nam NV (2005). Seroprevalence of dengue antibodies, annual incidence and risk factors among children in southern Vietnam. Tropical Med Int Health.

[79] World Health Organization (2019). Dengue control: the mosquito.

[80] López MAL, Dávila M, Canet M, López Y, Flores E, Dávila A (2017). Distribución de Aedes aegypti y Aedes albopictus en Guatemala 2016. Cienc Tecnol Salud.

[81] Clark-Gil S, Darsie R (1983). The mosquitoes of Guatemala, their identification, distribution and bionomics, with keys to adult females and larvae. Mosq Syst.

[82] Pérez Esquivel WA. Estudio sobre la prevalencia de casos positivos de enfermedades metaxénicas, en la población de Coatepeque, Quetzaltenango, de Enero a Octubre del 2016 según registros del Ministerio de Salud Pública y Asistencia Social Galileo Universidad 2019 [cited 2019 April 11]. Available from: http://biblioteca.galileo.edu/tesario/handle/123456789/761.

[83] Arevalo-Herrera M, Quiñones ML, Guerra C, Céspedes N, Giron S, Ahumada M (2012). Malaria in selected non-Amazonian countries of Latin America. Acta Trop.

[84] Kraemer MU, Sinka ME, Duda KA, Mylne AQ, Shearer FM, Barker CM (2015). The global distribution of the arbovirus vectors Aedes aegypti and Ae albopictus. Elife.

[85] Centers for Disease Control and Prevention (2018). About Malaria: Anopheles Mosquitoes.

[86] Nchoutpouen E, Talipouo A, Djiappi-Tchamen B, Djamouko-Djonkam L, Kopya E, Ngadjeu CS (2019). Culex species diversity, susceptibility to insecticides and role as potential vector of lymphatic filariasis in the city of Yaoundé, Cameroon. PLoS Negl Trop Dis.

[87] United Nations (2015). World population prospects: the 2015 revision. United Nations Dep Econ Soc Aff.

[88] Kraemer MU, Reiner RC, Brady OJ, Messina JP, Gilbert M, Pigott DM (2019). Past and future spread of the arbovirus vectors Aedes aegypti and Aedes albopictus. Nat Microbiol.

[89] Franklinos LH, Jones KE, Redding DW, Abubakar I (2019). The effect of global change on mosquito-borne disease. Lancet Infect Dis.

