# Supplementary material for: Associations between household environmental factors and immature mosquito abundance in Quetzaltenango, Guatemala
Source: BMC Public Health. 2019 Dec 23;19:1729. doi: 10.1186/s12889-019-8102-5 (PMC6929347; doi:10.1186/s12889-019-8102-5)
Supplement: Supplementary file 1 — Additional file 1 Table S1. Principal components factor analysis of household environment variables, Coatepeque and Génova, Guatemala, 2017 (n = 508). Table S2. Full model output of adjusted associations between geographical distances to paved roads and immature mosquito abundance, Poisson regression, Coatepeque and Génova, Guatemala, 2017. Table S3. Full model output of adjusted associations between geographical distances to highways and immature mosquito abundance, Poisson regression, Coatepeque and Génova, Guatemala, 2017. Table S4. Full model output of adjusted associations between geographical distances to houses or structures and immature mosquito abundance, Poisson regression, Coatepeque and Génova, Guatemala, 2017. Table S5. Associations between geographical distances to roads/structures and immature mosquito abundance, negative binomial regression, Coatepeque and Génova, Guatemala, 2017. Table S6. Mediation of distances to roads/structures and mosquito prevention measures on the association between environmental capital and the number of containers with any mosquito larvae or pupae per household, Coatepeque and Génova, Guatemala, 2017. Figure S1. Aerial view of communities in Coatepeque and Génova, Guatemala, 2017. Figure S2. Directed acyclic graphs of associations between geographical distances to roads and houses/structures and immature mosquito abundance. Figure S3. Cubic splines of associations between environmental capital and the number of containers with any mosquito larvae or pupae per household, Coatepeque and Génova, Guatemala, 2017. [file 12889_2019_8102_MOESM1_ESM.docx]

| **Table S1. Principal components factor analysis of household environment variables, Coatepeque and Génova, Guatemala, 2017 (n=508)** | |
| --- | --- |
| Characteristic | Factor pattern |
| Electricity | 0.31 |
| Running water | 0.71 |
| Television | 0.43 |
| Landline telephone | 0.35 |
| No pit latrine | 0.41 |
| Cable television | 0.65 |
| Garbage service | 0.72 |
| No water well | 0.60 |
| Sewer system | 0.72 |
| Number of rooms in house | 0.43 |
| *Eigenvalue* | 3.16 |

| **Table S2. Full model output^a^ of adjusted^b^ associations between geographical distances to paved roads and immature mosquito abundance, Poisson regression, Coatepeque and Génova, Guatemala, 2017.** | | | | | | | | | |
| --- | --- | --- | --- | --- | --- | --- | --- | --- | --- |
|  | Total number of larvae per household | | | Total number of pupae per household | | | Number of positive containers per household | | |
| Variable | β | SE | P-value | β | SE | P-value | β | SE | P-value |
| Distance from nearest paved road (10-m increase) | -0.04 | 0.01 | <0.01 | -0.07 | 0.03 | <0.01 | -0.04 | 0.01 | <0.01 |
| Environmental capital (ref: middle) |  |  | 0.50 |  |  | 0.82 |  |  | 0.25 |
| Low | -0.03 | 0.11 |  | -0.10 | 0.24 |  | -0.06 | 0.11 |  |
| High | -0.13 | 0.11 |  | -0.14 | 0.34 |  | -0.18 | 0.11 |  |
| Survey period (ref: February-March) | 0.40 | 0.15 | 0.06 | 0.37 | 0.21 | 0.08 | 0.42 | 0.18 | 0.06 |
| Urban residence (ref: rural) | 0.18 | 0.11 | 0.11 | 0.01 | 0.24 | 0.99 | 0.24 | 0.11 | 0.03 |
| Number of people / household | -0.01 | 0.02 | 0.81 | 0.04 | 0.03 | 0.26 | -0.01 | 0.02 | 0.36 |
| Cleaned containers (ref: no) | -0.09 | 0.09 | 0.34 | 0.01 | 0.21 | 0.99 | -0.07 | 0.08 | 0.43 |
| Fumigation (ref: no) | -0.10 | 0.11 | 0.39 | -0.40 | 0.28 | 0.16 | -0.18 | 0.10 | 0.07 |
| Total number of containers | 0.13 | 0.02 | <0.01 | 0.23 | 0.04 | <0.01 | 0.22 | 0.02 | <0.01 |
| ^a^These estimates should be interpreted with caution, because the relationships between the covariates and outcomes are not adjusted for confounders. Westreich D, Greenland S. The table 2 fallacy: presenting and interpreting confounder and modifier coefficients. American Journal of Epidemiology. 2013;177(4):292-8.  ^b^Adjusted for the other variables listed in the model. Environmental capital was derived from principal components factor analysis and included: number of rooms in the household; presence of electricity, running water, a television, a landline telephone, cable, trash disposal, and sewer system; and absence of a water well and pit latrine. | | | | | | | | | |

| **Table S3. Full model output^a^ of adjusted^b^ associations between geographical distances to highways and immature mosquito abundance, Poisson regression, Coatepeque and Génova, Guatemala, 2017.** | | | | | | | | | |
| --- | --- | --- | --- | --- | --- | --- | --- | --- | --- |
|  | Total number of larvae per household | | | Total number of pupae per household | | | Number of positive containers per household | | |
| Variable | β | SE | P-value | β | SE | P-value | β | SE | P-value |
| Distance from nearest highway (100-m increase) | 0.01 | 0.01 | 0.55 | 0.01 | 0.01 | 0.35 | 0.01 | 0.01 | 0.47 |
| Environmental capital (ref: middle) |  |  | 0.38 |  |  | 0.12 |  |  | 0.32 |
| Low | -0.17 | 0.18 |  | -0.05 | 0.19 |  | -0.19 | 0.16 |  |
| High | -0.12 | 0.10 |  | -0.12 | 0.09 |  | -0.17 | 0.17 |  |
| Survey period (ref: February-March) | 0.28 | 0.12 | 0.10 | 0.23 | 0.10 | 0.17 | 0.25 | 0.15 | 0.20 |
| Urban residence (ref: rural) | 0.17 | 0.02 | <0.01 | -0.02 | 0.04 | 0.67 | -0.02 | 0.04 | 0.67 |
| Number of people / household | 0.01 | 0.01 | 0.02 | 0.02 | 0.02 | 0.30 | 0.02 | 0.02 | 0.18 |
| Cleaned containers (ref: no) | -0.07 | 0.06 | 0.22 | 0.03 | 0.03 | 0.41 | 0.03 | 0.03 | 0.41 |
| Fumigation (ref: no) | -0.08 | 0.06 | 0.18 | -0.28 | 0.11 | 0.15 | -0.22 | 0.09 | 0.06 |
| Total number of containers | 0.13 | 0.02 | <0.01 | 0.20 | 0.03 | <0.01 | 0.18 | 0.03 | <0.01 |
| ^a^These estimates should be interpreted with caution, because the relationships between the covariates and outcomes are not adjusted for confounders. Westreich D, Greenland S. The table 2 fallacy: presenting and interpreting confounder and modifier coefficients. American Journal of Epidemiology. 2013;177(4):292-8.  ^b^Adjusted for the other variables listed in the model. Environmental capital was derived from principal components factor analysis and included: number of rooms in the household; presence of electricity, running water, a television, a landline telephone, cable, trash disposal, and sewer system; and absence of a water well and pit latrine. | | | | | | | | | |

| **Table S4. Full model output^a^ of adjusted^b^ associations between geographical distances to houses or structures and immature mosquito abundance, Poisson regression, Coatepeque and Génova, Guatemala, 2017.** | | | | | | | | | |
| --- | --- | --- | --- | --- | --- | --- | --- | --- | --- |
|  | Total number of larvae per household | | | Total number of pupae per household | | | Number of positive containers per household | | |
| Variable | β | SE | P-value | β | SE | P-value | β | SE | P-value |
| Distance from nearest structure (1-m increase) | -0.03 | 0.01 | <0.01 | -0.05 | 0.02 | 0.02 | -0.03 | 0.01 | <0.01 |
| Environmental capital (ref: middle) |  |  | 0.50 |  |  | 0.83 |  |  | 0.25 |
| Low | -0.07 | 0.12 |  | -0.11 | 0.25 |  | -0.09 | 0.12 |  |
| High | -0.13 | 0.11 |  | -0.13 | 0.35 |  | -0.18 | 0.11 |  |
| Survey period (ref: February-March) | 0.41 | 0.12 | 0.06 | 0.34 | 0.22 | 0.09 | 0.38 | 0.20 | 0.08 |
| Urban residence (ref: rural) | 0.15 | 0.11 | 0.18 | -0.03 | 0.25 | 0.90 | 0.22 | 0.11 | 0.06 |
| Number of people / household | -0.01 | 0.02 | 0.68 | 0.03 | 0.03 | 0.33 | -0.02 | 0.02 | 0.29 |
| Cleaned containers (ref: no) | -0.07 | 0.09 | 0.49 | 0.04 | 0.22 | 0.87 | -0.05 | 0.08 | 0.59 |
| Fumigation (ref: no) | -0.09 | 0.11 | 0.42 | -0.39 | 0.28 | 0.17 | -0.18 | 0.10 | 0.08 |
| Total number of containers | 0.14 | 0.02 | <0.01 | 0.24 | 0.03 | <0.01 | 0.23 | 0.02 | <0.01 |
| ^a^These estimates should be interpreted with caution, because the relationships between the covariates and outcomes are not adjusted for confounders. Westreich D, Greenland S. The table 2 fallacy: presenting and interpreting confounder and modifier coefficients. American Journal of Epidemiology. 2013;177(4):292-8.  ^b^Adjusted for the other variables listed in the model. Environmental capital was derived from principal components factor analysis and included: number of rooms in the household; presence of electricity, running water, a television, a landline telephone, cable, trash disposal, and sewer system; and absence of a water well and pit latrine. | | | | | | | | | |

| **Table S5. Associations between geographical distances to roads/structures and immature mosquito abundance, negative binomial regression, Coatepeque and Génova, Guatemala, 2017.** | | | | | | | | | |
| --- | --- | --- | --- | --- | --- | --- | --- | --- | --- |
|  | Total number of larvae per household | | | Total number of pupae per household | | | Number of positive containers per household | | |
| Variable | β | SE | P-value | β | SE | P-value | β | SE | P-value |
| Distance from nearest paved road (10-m increase) |  |  |  |  |  |  |  |  |  |
| Unadjusted | -0.04 | 0.01 | <0.01 | -0.06 | 0.02 | <0.01 | -0.04 | 0.01 | <0.01 |
| Adjusted^a^ | -0.04 | 0.01 | <0.01 | -0.08 | 0.02 | <0.01 | -0.04 | 0.01 | <0.01 |
| Distance from nearest highway (100-m increase) |  |  |  |  |  |  |  |  |  |
| Unadjusted | 0.01 | 0.01 | 0.49 | 0.01 | 0.02 | 0.27 | 0.02 | 0.01 | 0.41 |
| Adjusted^a^ | -0.01 | 0.01 | 0.73 | 0.02 | 0.02 | 0.64 | 0.01 | 0.01 | 0.78 |
| Distance from nearest structure (1-m increase) |  |  |  |  |  |  |  |  |  |
| Unadjusted | -0.02 | 0.01 | <0.01 | -0.06 | 0.01 | <0.01 | -0.04 | 0.01 | <0.01 |
| Adjusted^a^ | -0.02 | 0.01 | <0.01 | -0.06 | 0.02 | <0.01 | -0.03 | 0.01 | <0.01 |
| ^a^Adjusted for environmental capital, survey period, urban/rural residence, the number of people in a household, cleaned containers, fumigated inside or outside the house, and the total number of containers. Environmental capital was derived from principal components factor analysis and included: number of rooms in the household; presence of electricity, running water, a television, a landline telephone, cable, trash disposal, and sewer system; and absence of a water well and pit latrine. | | | | | | | | | |

| **Table S6. Mediation of distances to roads/structures and mosquito prevention measures on the association between environmental capital and the number of containers with any mosquito larvae or pupae per household, Coatepeque and Génova, Guatemala, 2017** | | | | | | | | | | |  |
| --- | --- | --- | --- | --- | --- | --- | --- | --- | --- | --- | --- |
|  | Controlled direct effect | | Natural indirect effect | | | Total effect | | |  | |  |
| Characteristic | Estimate | 95% CI | | Estimate | 95% CI | | Estimate | 95% CI | | Proportion mediated | |
| Fumigated house | 0.08* | 0.05, 0.11 | | -0.01 | -0.02, 0.01 | | 0.08* | 0.04, 0.11 | | -0.08 | |
| Cleaned containers | 0.08* | 0.05, 0.11 | | -0.01 | -0.02, 0.01 | | 0.07* | 0.04, 0.11 | | -0.04 | |
| Distance to paved road (m) | 0.01 | -0.02, 0.04 | | 0.22* | 0.16, 0.29 | | 0.24* | 0.18, 0.28 | | 0.94 | |
| Distance to highway (m) | 0.08* | 0.04, 0.10 | | -0.01 | -0.03, 0.01 | | 0.07* | 0.04, 0.10 | | -0.02 | |
| Distance to nearest structure (m) | 0.03 | 0.03, -0.01 | | 0.12* | 0.06, 0.18 | | 0.15* | 0.10, 0.19 | | 0.80 | |
| *p-value<0.05 | | | | | | | | | | |  |

**Figure S1. Aerial view of communities in Coatepeque and Génova, Guatemala, 2017.** The study sites are enclosed in red. Source: Quetzaltenango department location map; by user Edouno; licensed under CC BY 3.0 via Wikimedia Commons, https://commons.wikimedia.org/wiki/File:Quetzaltenango_department_location_map.svg.


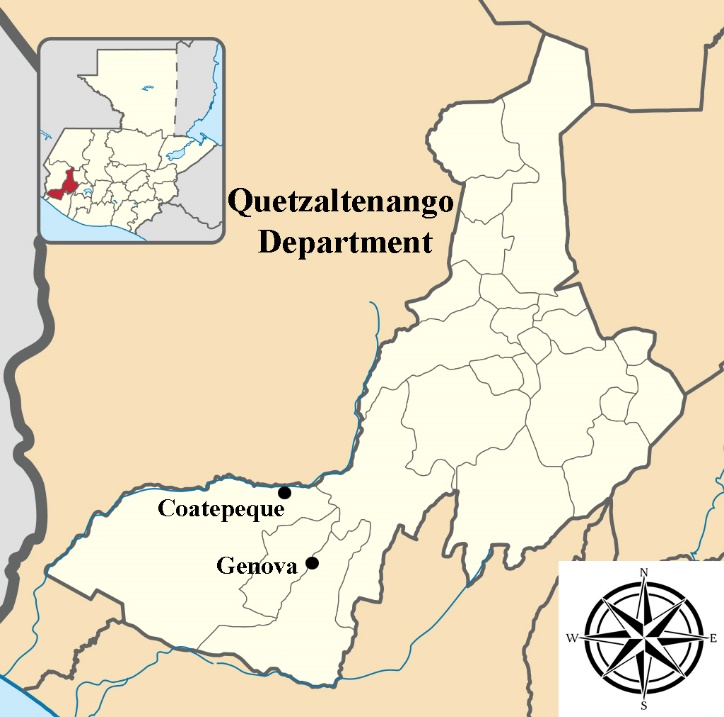


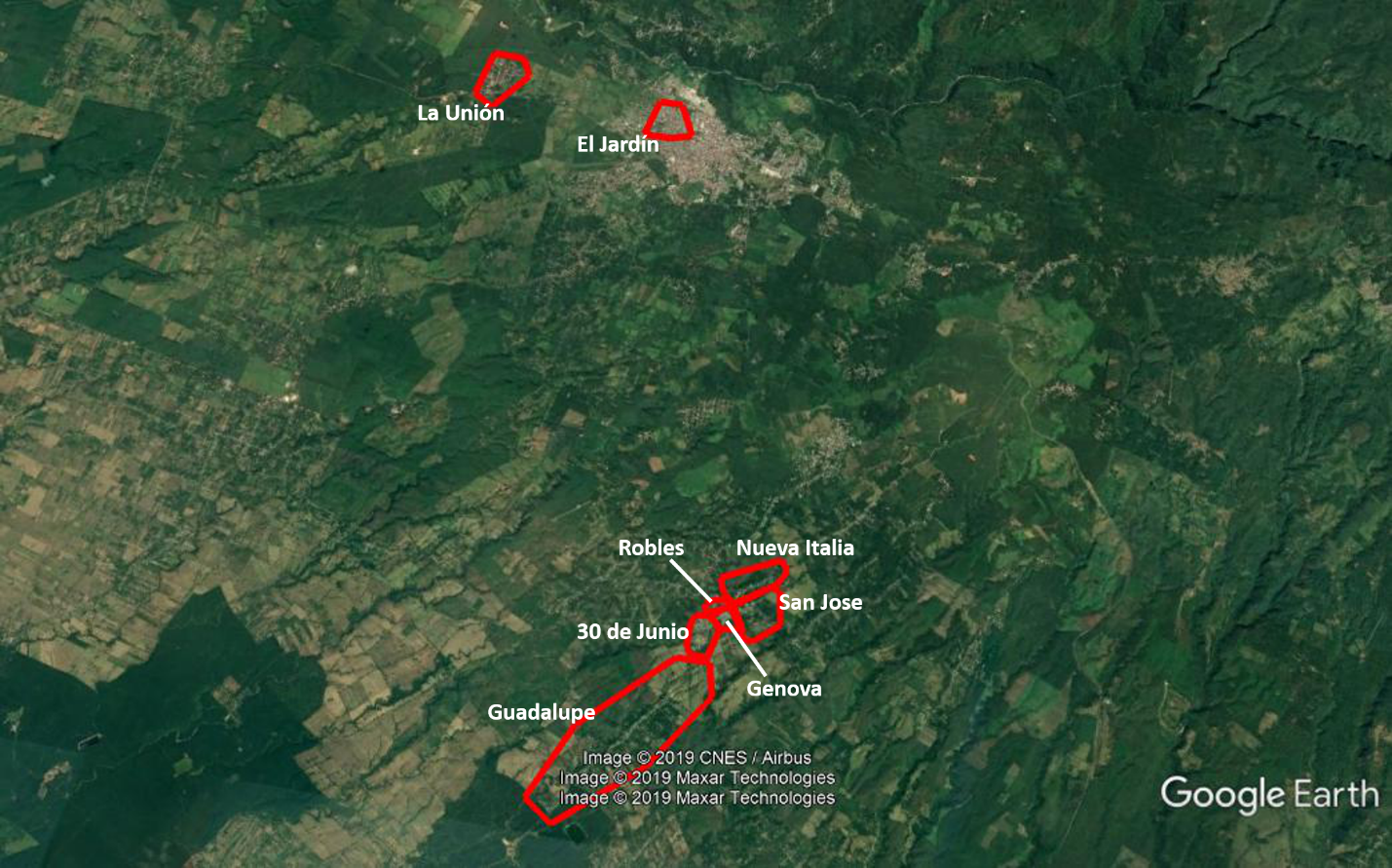


**Figure S2. Directed acyclic graphs of associations between geographical distances to roads and houses/structures and immature mosquito abundance.** Panel A: distance to paved roads; Panel B: distance to highways; Panel C: distance to nearest house.

| Panel A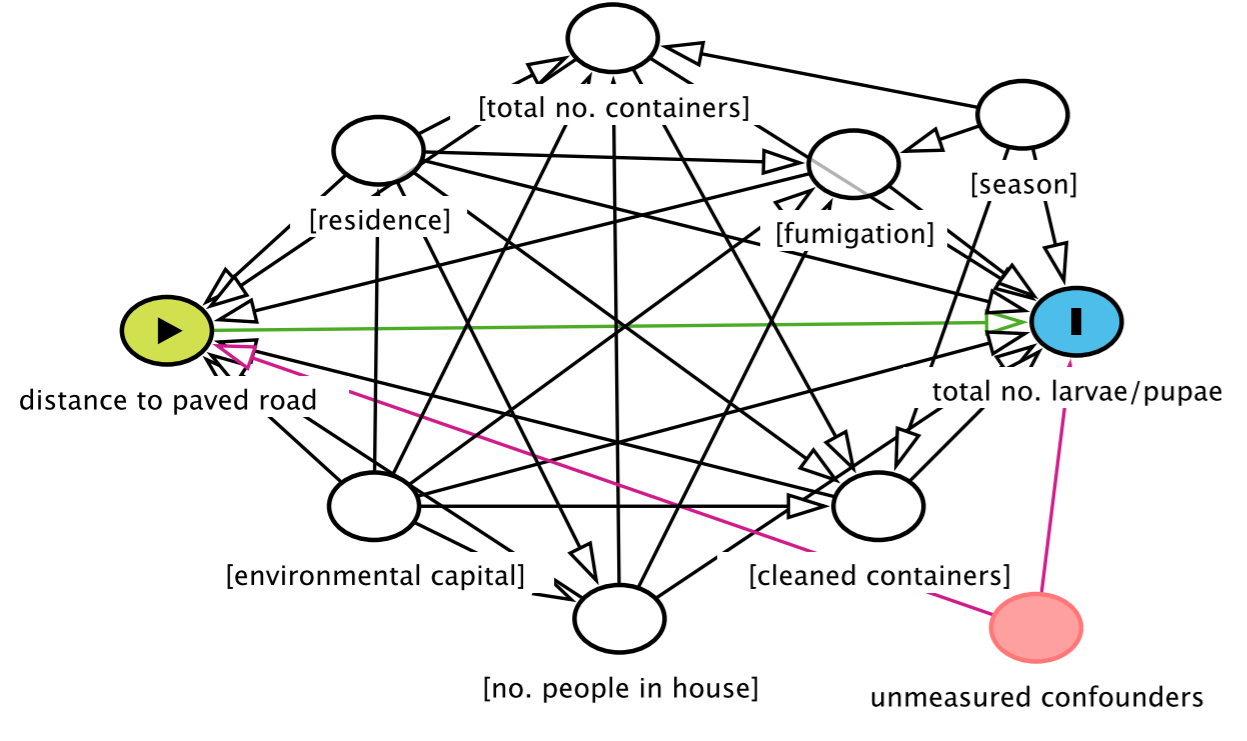 | Panel B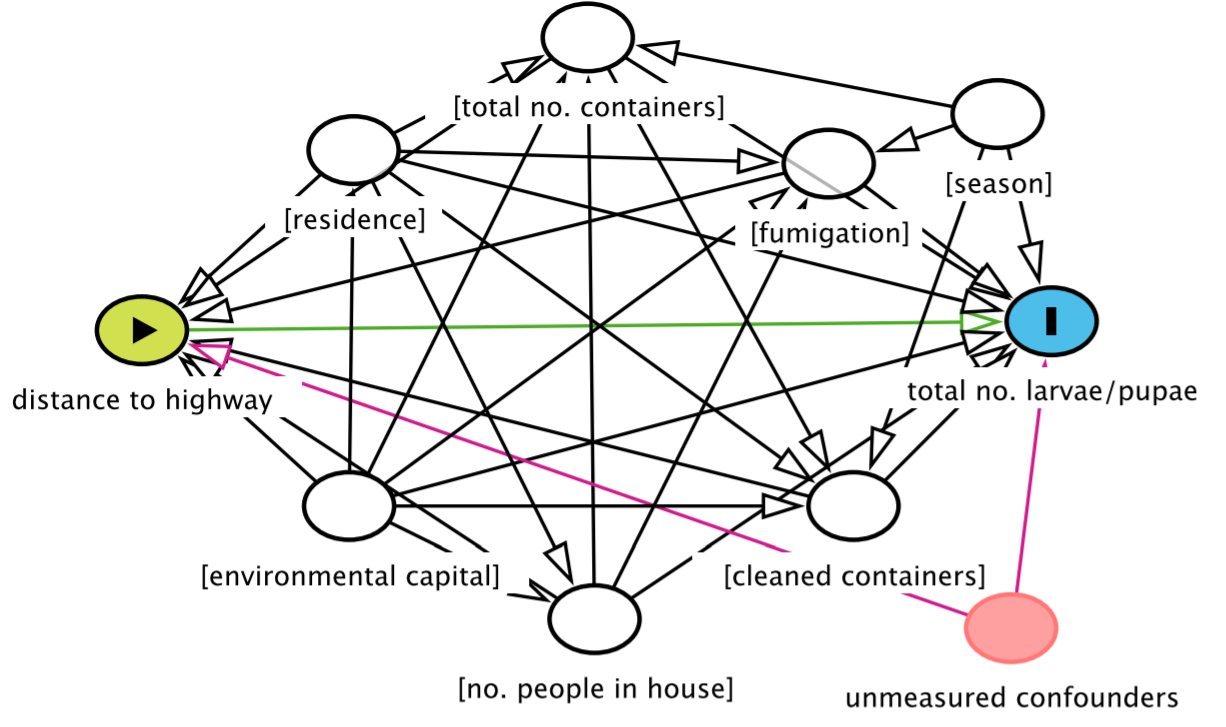 |
| --- | --- |
| Panel C  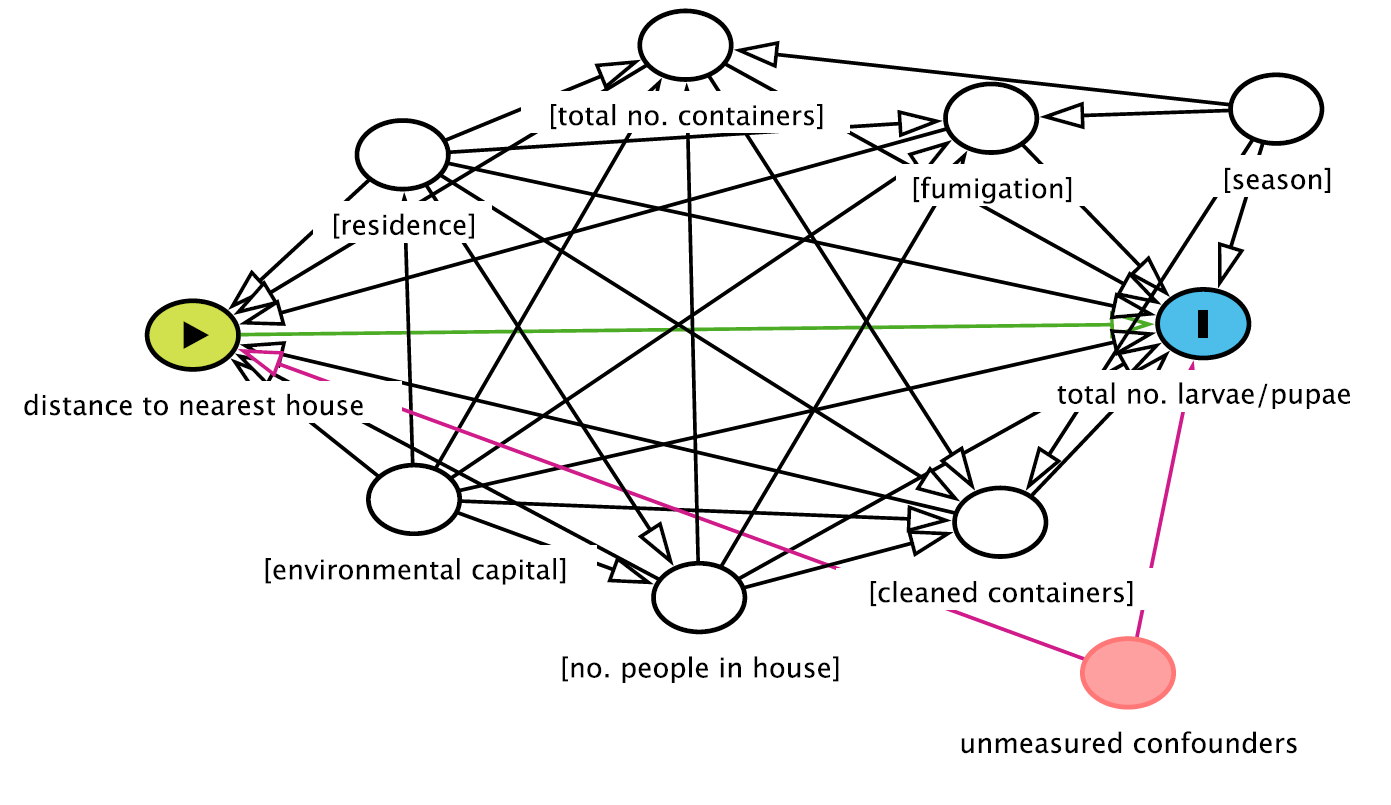 | |

**Figure S3. Cubic splines of associations between environmental capital and the number of containers with any mosquito larvae or pupae per household, Coatepeque and Génova, Guatemala, 2017**. Panel A shows results from the first survey in February-March, 2017. Panel B shows results from the second survey in November-December, 2017. The bands represent 95% confidence intervals.

| Panel A  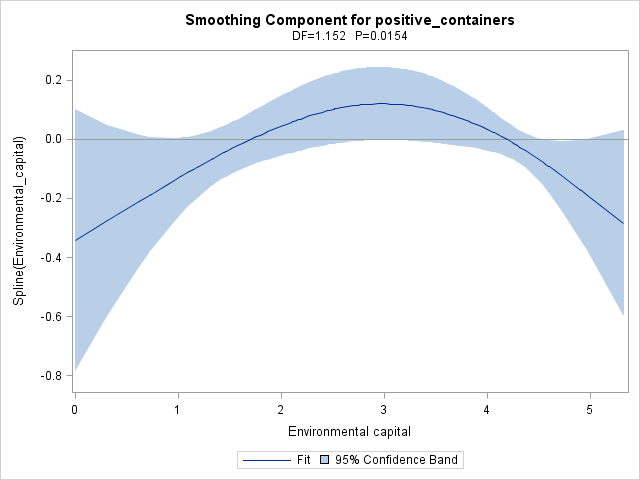 | Panel B  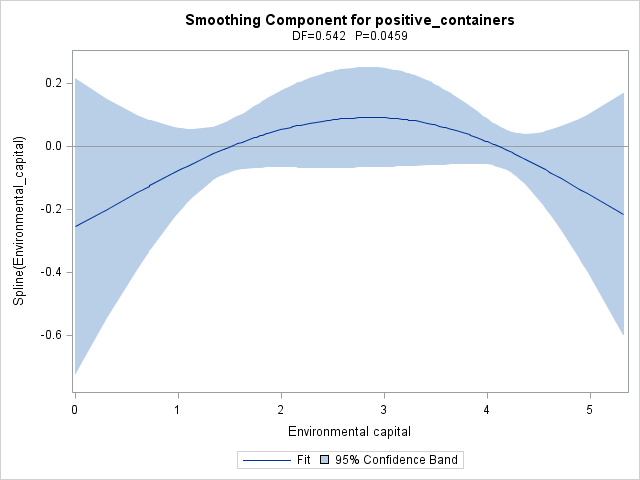 |
| --- | --- |
